# Origin variations and brachial plexus relationship of the dorsal scapular artery

**DOI:** 10.1038/s41598-023-35054-2

**Published:** 2023-05-13

**Authors:** Kuen-Cherng Lai, Han-Chen Ho

**Affiliations:** Department of Anatomy, Tzu Chi University, 701, Section 3, Chung-Yang Road, Hualien, 970374 Taiwan

**Keywords:** Anatomy, Medical research

## Abstract

The dorsal scapular artery can either be a direct branch of the subclavian artery or a branch of the transverse cervical artery. Origin variation is related to its relationship with the brachial plexus. Anatomical dissection was performed on 79 sides of 41 formalin-embalmed cadavers in Taiwan. The origin of the dorsal scapular artery and the variations of its brachial plexus relationship were scrutinized and analyzed. Results showed that the dorsal scapular artery originated most frequently from the transverse cervical artery (48%), followed by the direct branch from the third part (25%) and the second part (22%) of the subclavian artery and from the axillary artery (5%). Only 3% of the dorsal scapular artery passed through the brachial plexus if its origin was the transverse cervical artery. However, 100% and 75% of the dorsal scapular artery passed through the brachial plexus when they were direct branches of the second and the third part of the subclavian artery, respectively. Suprascapular arteries were also found to pass through the brachial plexus when they were direct branches from the subclavian artery, but all passed over or under the brachial plexus if they originated from the thyrocervical trunk or transverse cervical artery. Variations in the origin and course of arteries around the brachial plexus are of immense value not only to the basic anatomical knowledge but also to clinical practices such as supraclavicular brachial plexus block and head and neck reconstruction with pedicled or free flaps.

## Introduction

The subclavian artery supplies blood to the upper limbs and parts of the head and neck. It is divided into three parts: the first is medial to the anterior scalene muscle, the second is posterior to the anterior scalene muscle, and the third is lateral to the anterior scalene muscle^1^. Often considered a direct branch of the subclavian artery^2^, the dorsal scapular artery (DSA) supplies the rhomboids, levator scapulae, and the lower part of the trapezius muscles. However, a common variation of the origin of the DSA is also described as the deep branch of the transverse cervical artery (TCA) arising from the thyrocervical trunk^2,3^. The predominant origin site of the DSA varied among studies. Studies from the United States, France, Germany, Spain, etc., reviewed by Huelke^4^, found that the DSA frequently originated approximately 70% of the time from the subclavian artery, either from the second or the third part. However, studies in Japan and Thailand showed that the DSA originated more often from the TCA (55.7% in Japan and 69% in Thailand) than from the subclavian artery^5,6^.

The DSA courses will be different depending on its site of origin. The relationship of the DSA to the nearby brachial plexus is of concern. Reports showed that the DSA passed through the brachial plexus if it originated from the subclavian artery^2,6^. In clinical practice, the supraclavicular brachial plexus block provides anesthesia and analgesia to the upper extremity below the shoulder, which is an excellent choice for elbow and hand surgeries. However, failure of the block was reported by the limited spread of local anesthetic because of blockage by vessels running through the brachial plexus^7^. Arterial compression of the brachial plexus might also result in pain or related thoracic outlet syndrome, which is characterized by pain and paresthesia in the upper limb^8^.

Our goal was to conduct a survey of the variations of DSA origin and the relationship of the route of the DSA to the brachial plexus in cadavers in Taiwan. Identification of the anatomical variation will improve the safety and quality of clinical practices such as supraclavicular brachial plexus block and head and neck reconstruction with pedicled or free flaps.

## Materials and methods

This study was conducted in the Gross Anatomy Laboratory in the Department of Anatomy at Tzu Chi University (Hualien, Taiwan). A total of 41 formalin-embalmed cadavers (20 females and 21 males) were dissected, with an age range of 47–92 (68.56 ± 12.61) years. The Tzu Chi Willed Body Donation Program, which is dedicated to medical education, obtained all the cadavers with informed consents. Each donor with two of his/her relatives co-signed the willed body donation form. This study was approved by the ethics committee of the Willed Body Donation Program of the Tzu Chi University. The authors hereby confirm that every effort was made to comply with all local and international ethical guidelines and laws concerning the use of human cadaveric donors in anatomical research.

At the end of the medical gross anatomy courses, the branches of the subclavian artery were carefully traced toward the muscles for accurate identification, and 79 sides (39 left and 40 right sides, 37 females and 42 males) were exposed for evaluation. Three sides were unable to evaluate because of invading tumors and fibrosis. The variations of origin of the DSA and its relationship to the brachial plexus were recorded on all cadavers by the same investigator.

Data were presented as absolute numbers and percentages.

## Results

The DSA originated more frequently from the TCA (38/79, 48%) than from the second part (22%) or the third part (25%) of the subclavian artery, or the second and the third parts combined (47%) (Table 1). Additionally, 5% of the DSA originated from the axillary artery, derived from the third part of the subclavian artery. The frequency of DSA originating from the third part of the subclavian artery appeared higher in males than in females.

**Table 1:** Variations of origin of the dorsal scapular artery (n = 79).

Origin	Male	Female	Total	Percentage (%)
Transverse cervical a.	17	21	38	48
Subclavian II	8	9	17	22
Subclavian III	16	4	20	25
Axillary a.	1	3	4	5

Subclavian II, the second part of the subclavian artery; Subclavian III, the third part of the subclavian artery.

### DSA originated from the TCA rarely passed through the brachial plexus

**Figure 1:**
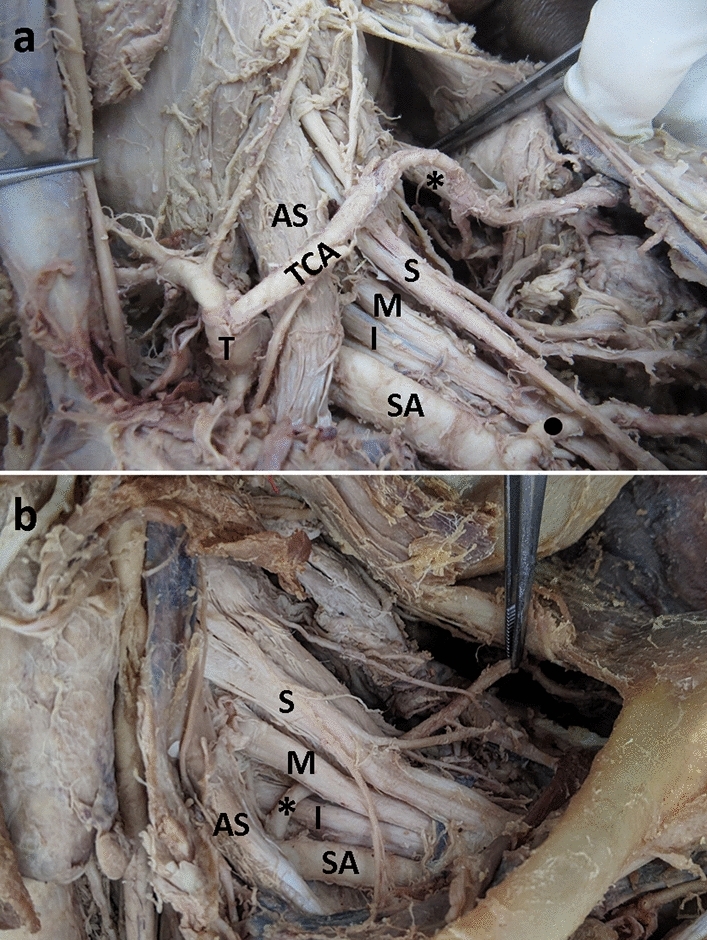
Representative images showing the relationships of the brachial plexus and arteries. (**a**) Dorsal scapular artery (*) is a branch of the transverse cervical artery (TCA), which arises from the thyrocervical trunk (T). The above arteries pass over the brachial plexus towards the back. The suprascapular artery (●) passing through the brachial plexus is a branch of the axillary artery. AS: anterior scalene muscle; I: inferior trunk; M: middle trunk; S: superior trunk of brachial plexus; SA: subclavian artery. (**b**) Dorsal scapular artery (*) passing through the middle (M) and inferior (I) trunks of the brachial plexus is a direct branch from the second part of the subclavian artery (SA).

For the 38 DSAs that originated from the TCAs, 31 of the TCAs were derived from the thyrocervical trunk and all of them passed over the brachial plexus (Fig. 1a). Of the other seven TCAs that gave rise to the DSAs, four were found to be direct branches from the first part of the subclavian artery, one from the second part of the subclavian artery, and two from the third part of the subclavian artery. The paths of the TCA/DSA were related to their origins. The ones that derived from the first part of the subclavian artery all passed over the brachial plexus. The two that arose from the third part of the subclavian artery passed under the brachial plexus. Only the one that originated from the second part of the subclavian artery ran through the brachial plexus between the middle and inferior trunks (Table 2).

**Table 2:** Origin of the transverse cervical artery that give rise to the dorsal scapular artery and the relationship to the brachial plexus (n = 38).

Origin	Male	Female	Total	Relationship to the brachial plexus
Thyrocervical trunk	12	19	31	100% (31/31) pass over the brachial plexus
Subclavian I	3	1	4	100% (4/4) pass over the brachial plexus
Subclavian II	0	1	1	Between the middle and inferior trunks
Subclavian III	2	0	2	100% (2/2) pass under the brachial plexus

Subclavian I, the first part of the subclavian artery; Subclavian II, the second part of the subclavian artery; Subclavian III, the third part of the subclavian artery.

### DSA originated from the subclavian artery tend to pass through the brachial plexus

On the contrary, 100% of the DSAs that directly arose from the second part of the subclavian artery passed through the brachial plexus (Fig. 1b) (Table 3). The majority of the DSAs passed through the middle and inferior trunks of the brachial plexus (10/17, 59%), whereas 35% (6/17) of the DSAs passed through the superior and middle trunks of the brachial plexus, and one (6%) passed through the C8 and T1 roots. The DSAs that arose directly from the third part of the subclavian artery showed 40% (8/20) of the arteries passed through the middle and inferior trunks, whereas 35% (7/20) passed through the superior and middle trunks of the brachial plexus, and 25% (5/20) passed under the brachial plexus. We also found that of the four DSAs that arose from the axillary arteries, two (50%) of them passed through the superior and middle trunks of the brachial plexus and two (50%) of them passed under the brachial plexus.

**Table 3:** Relationships of the DSAs arise from the subclavian artery or axillary artery to the brachial plexus (n = 41).

Origins and paths of the DSAs	Male	Female	Total	% Pass through the brachial plexus
**Subclavian II**	**8**	**9**	**17**	**100% (17/17)**
Between the superior and middle trunks	4	2	6	35% (6/17)
Between the middle and inferior trunks	4	6	10	59% (10/17)
Between C8 and T1 roots	0	1	1	6% (1/17)
**Subclavian III**	**16**	**4**	**20**	**75% (15/20)**
Between the superior and middle trunks	5	2	7	35% (7/20)
Between the middle and inferior trunks	7	1	8	40% (8/20)
Pass under the brachial plexus	4	1	5	–
**Axillary artery**	**1**	**3**	**4**	**50% (2/4)**
Between the superior and middle trunks	1	1	2	50% (2/4)
Pass under the brachial plexus	0	2	2	–

Subclavian II, the second part of the subclavian artery; Subclavian III, the third part of the subclavian artery. Three main groups and corresponding numbers are in bold.

### Suprascapular artery showed similar trend as the DSA

While investigating the routes of the DSAs, we found that some suprascapular arteries also passed through the brachial plexus (Fig. 1). A total of 15 sides showed both DSA and suprascapular artery within the brachial plexus. We identified 72 suprascapular arteries where the paths were clearly traced toward the supraspinatus. The suprascapular arteries all passed over or under the brachial plexus without passing through it (47/72, 65%) when originating from the thyrocervical trunk or the TCA. However, 96% (24/25) of them passed through the brachial plexus (Table 4) if the suprascapular arteries originated directly from the subclavian artery or the axillary artery. Additionally, 50% (12/24) of them passed through the divisions downstream to the superior and middle trunks of the brachial plexus (Table 4). Taken together, 44% (35/79) of the DSAs passed through the brachial plexus and 33% (24/72) of the suprascapular arteries passed through the brachial plexus in this study. The DSAs passed most frequently through the middle and inferior trunks of the brachial plexus, while the suprascapular artery passed predominantly between the divisions downstream to the superior and middle trunks of the brachial plexus.

**Table 4:** Relationships of the suprascapular artery to the brachial plexus (n = 72).

Origins and paths of the suprascapular artery	Number	Relationship to the brachial plexus
**Thyrocervical trunk or transverse cervical artery**	**47**	100% (47/47) pass over or under the brachial plexus
**Subclavian II**	**3**	100% (3/3) pass through the brachial plexus
Between the superior and middle trunks	1	
Between the middle and inferior trunks	2	
**Subclavian III**	**9**	100% (9/9) pass through the brachial plexus
Between the superior and middle trunks	4	
Between the divisions downstream to the superior and middle trunks	5	
**Axillary artery**	**13**	92% (12/13) pass through the brachial plexus
Between the superior and middle trunks	5	
Between the divisions downstream to the superior and middle trunks	7	
Pass under the brachial plexus	1	

Subclavian II, the second part of the subclavian artery; Subclavian III, the third part of the subclavian artery.

Four main groups and corresponding numbers are in bold.

Interestingly, our data revealed that both DSA and suprascapular artery originating from the subclavian artery had significantly higher rates passing through the brachial plexus than originating from the thyrocervical trunk/TCA.

## Discussion

Our study focusing on the Taiwanese population revealed that the DSA originated from the TCA (48%) more often than from the second and the third parts of the subclavian artery combined (47%). This is similar to results with a higher percentage of DSA originating from the TCA found in studies in Japan (55.7%)^5^ and Thailand (69%)^6^. Contrary to studies in Asia, DSAs were reported to originate more frequently from the subclavian artery in the United States (61.2% in^2^, 74.6% in^3^, and 71% in^8^). Earlier studies from France, Germany, Italy, Hungary, Switzerland, and Spain also showed that the DSA originated predominantly from the subclavian artery^4^. Although it is not clear whether the differences in DSA origin is related to race, it is important for physicians to be informed of these variations when performing procedures in their respective regions.

Although the overall percentages of DSA passing through the brachial plexus was only 44% in the Taiwanese population, 100% and 75% of the DSAs were found passing through the brachial plexus when they originated directly from the second and the third parts of the subclavian artery, respectively. Limited studies investigating the relationships between the DSA and brachial plexus also reported similar results, with > 90% of the DSA passing through the brachial plexus when they were direct branches from the subclavian artery^2,6^. The close apposition of the subclavian artery and brachial plexus could be the reason for the high incidence of DSA passing through the brachial plexus.

The presence of vessels within the brachial plexus may pose a risk to health. Arterial compression of the brachial plexus might result in pain or related thoracic outlet syndrome. Verenna et al.^8^ reported a patient diagnosed with thoracic outlet syndrome, and symptoms were not relieved until the DSA, which was a direct branch from the subclavian artery and passed through the brachial plexus, was surgically ligated. Therefore, in case of upper extremity radiating pain or paresthesia with obvious proximal etiology, the possibility of a vascular compression of the brachial plexus should be considered^8^. Moreover, the presence of vessels within the brachial plexus may affect the safety and efficacy of brachial plexus blockade^7^. Vascular puncture-related complications may threaten the safety of the patients; however, sonographic assessment can significantly reduce such complications^9^. Nambyiah et al.^10^ had successfully identified arteries within the brachial plexus in > 90% of subjects using a portable ultrasound device.

In addition to DSA, the suprascapular artery was also found passing through the brachial plexus in our study. The overall percentage for the suprascapular arteries passing through the brachial plexus was 33%, similar to results found in France (28%)^11^. However, 100% and 92% of the suprascapular arteries were found within the brachial plexus when they were direct branches from the subclavian or axillary arteries, respectively. Moreover, 15 sides of the brachial plexus examined in our study had both the DSA and suprascapular artery passing through brachial plexus, which may pose higher risks for neurovascular complications around this region.

Variations in the origin and course of arteries around the brachial plexus are of immense value to surgeons, angiographists, and anatomists. For example, the pedicled DSA flaps are widely used for reconstruction of head, neck, and upper back defects^12,13^. Sufficient perfusion in the distal region determined the flap survival rates and therefore the outcome of the surgery. In this study, we showed that DSAs and the suprascapular artery originating directly from the subclavian artery had high percentages running through the brachial plexus, which were reported to impede needle approaches and may cause technical difficulties or complications in supraclavicular blocks^7,14^. Although there is no information about whether the perfusions of arteries were affected when they passed through the brachial plexus, we believe it would be beneficial and important to acquire this information for clinical evaluation.

## Conclusion

The DSA is predominantly originated from the TCA instead of the subclavian artery in Taiwanese population. When the artery is a direct branch of the subclavian artery, it has a higher incidence of passing through the brachial plexus. A sonographic assessment around this region before surgeries could be advantageous in reducing neurovascular complications.

## Data Availability

Data are available from the corresponding author (H.-C. Ho) upon request.
